# BCL-2 Proteins in Pathogenesis and Therapy of B-Cell Non-Hodgkin Lymphomas

**DOI:** 10.3390/cancers12040938

**Published:** 2020-04-10

**Authors:** Magdalena Klanova, Pavel Klener

**Affiliations:** 1 Institute of Pathological Physiology, First Faculty of Medicine, Charles University in Prague, 12853 Prague, Czech Republic; pavel.klener2@lf1.cuni.cz; 2 First Department of Internal Medicine—Department of Hematology, Charles University General Hospital in Prague, 12808 Prague, Czech Republic

**Keywords:** apoptosis, non-Hodgkin lymphomas (NHL), B-cell leukemia/lymphoma-2 (BCL-2), venetoclax

## Abstract

The ability to inhibit mitochondrial apoptosis is a hallmark of B-cell non-Hodgkin lymphomas (B-NHL). Activation of mitochondrial apoptosis is tightly controlled by members of B-cell leukemia/lymphoma-2 (BCL-2) family proteins via protein-protein interactions. Altering the balance between anti-apoptotic and pro-apoptotic BCL-2 proteins leads to apoptosis evasion and extended survival of malignant cells. The pro-survival BCL-2 proteins: B-cell leukemia/lymphoma-2 (BCL-2/BCL2), myeloid cell leukemia-1 (MCL-1/MCL1) and B-cell lymphoma-extra large (BCL-XL/BCL2L1) are frequently (over)expressed in B-NHL, which plays a crucial role in lymphoma pathogenesis, disease progression, and drug resistance. The efforts to develop inhibitors of anti-apoptotic BCL-2 proteins have been underway for several decades and molecules targeting anti-apoptotic BCL-2 proteins are in various stages of clinical testing. Venetoclax is a highly specific BCL-2 inhibitor, which has been approved by the US Food and Drug Agency (FDA) for the treatment of patients with chronic lymphocytic leukemia (CLL) and is in advanced clinical testing in other types of B-NHL. In this review, we summarize the biology of BCL-2 proteins and the mechanisms of how these proteins are deregulated in distinct B-NHL subtypes. We describe the mechanism of action of BH3-mimetics and the status of their clinical development in B-NHL. Finally, we summarize the mechanisms of sensitivity/resistance to venetoclax.

## 1. Introduction

**Figure 1:**
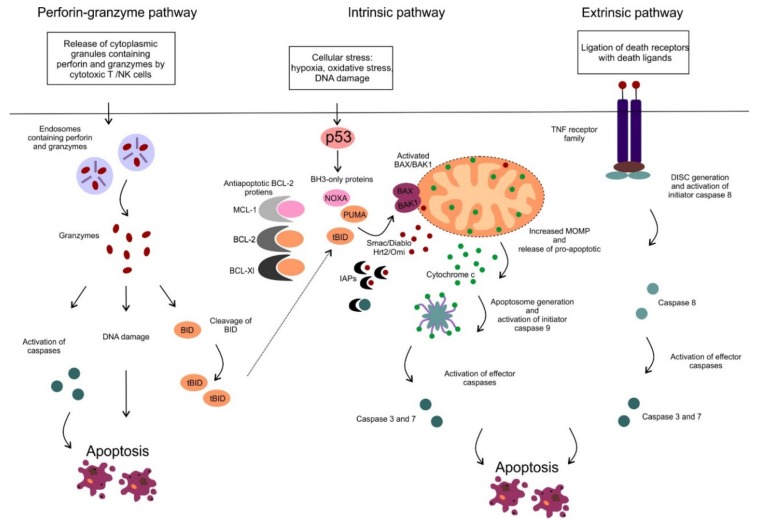
The perforin-granzyme, intrinsic (mitochondrial) and extrinsic apoptotic pathway. The extrinsic pathway is triggered via ligation of cell surface death receptors from the tumor necrosis factor (TNF) receptor family with death ligands (e.g., FAS ligand or TNFα) on the outside of the cell membrane. Subsequently, a multiprotein complex: death-inducing signaling complex (DISC) is created on the cytosolic side of the cell membrane resulting in activation of pro-caspase 8. The initiator caspase 8 subsequently activates the effector caspases (caspase-3 and 7), that cleave cellular proteins and promotes the apoptotic machinery. The perforin-granzyme and intrinsic apoptotic pathways are described in the text.

Apoptosis is an active form of programmed cell death, in which a controlled sequence of events leads to the elimination of aged, damaged and unnecessary cells [1]. The ability to block apoptosis is a hallmark of many cancers, including hematologic malignancies. Several other types of cell death have been described so far such as necrosis, necroptosis, pyroptosis, ferroptosis, oncosis or autophagy that differ from each other in the triggering process mediating cell death, the involved pathways and/or the impact on the surrounding microenvironment [2]. There are two main apoptotic pathways, the extrinsic or death receptor pathway and the intrinsic or mitochondrial pathway. The extrinsic pathway is triggered by ligation of cell surface death receptors of the tumor necrosis factor (TNF) superfamily, such as TNF receptor 1 or FAS, with specific death ligands [3]. On the contrary, the intrinsic or mitochondrial pathway is triggered by various cellular stresses such as hypoxia, oxidative stress or DNA damage (Figure 1).

The two main apoptotic pathways meet at the same execution step resulting in the activation of effector enzymes, cysteine-dependent aspartate-directed proteases, called caspases (Figure 1). Activated caspases possess proteolytic activity and can cleave proteins at aspartic acid residues and target cytoskeletal or nuclear proteins resulting in highly controlled cell disintegration. Another apoptotic pathway has been described, which is mediated by cytotoxic T cells and natural killer (NK) cells (Figure 1). These immune cells can effectively kill infected or transformed cells via the perforin-granzyme apoptotic pathway. Target cells are first exposed to perforin, the protein capable of creating pores in the plasma membrane. Subsequently, secretory granules containing granzyme A and B are released by the immune cells, entering the target cells through the pores. It has also been shown, that perforin and granzymes enter the target cell via receptor-mediated endocytosis (Figure 1) [4]. Granzyme A and B are serine proteases that can cleave proteins at aspartate residues, thus capable of activating caspases and triggering apoptosis [5]. Granzyme B also triggers caspase activation indirectly by activation of pro-apoptotic BH3-only proteins, such as BH3-interacting domain death agonist (BID) which results in the activation of the mitochondrial apoptotic pathway (in detail described below) [6]. In addition, granzyme A can also cleave nuclear proteins and induce single-strand DNA breaks (Figure 1) [7].

Apoptosis is accompanied by characteristic morphological changes of the dying cells. These morphological features are visible via light microscopy and typically include cell shrinkage and nuclear pyknosis caused by chromatin condensation. Another characteristic feature of apoptosis is plasma membrane blebbing with subsequent formation of apoptotic bodies consisting of cytoplasm, intact organelles and nuclear fragments [8]. The apoptotic bodies are cleared via phagocytes without releasing pro-inflammatory cell contents into the surrounding area.

## 2. Mitochondrial Apoptotic Pathway

In this review, we focus on the mitochondrial apoptotic pathway, which plays a critical role in pathogenesis and drug resistance of distinct hematologic malignancies and has been extensively studied and targeted therapeutically. The activation of the mitochondrial pathway leads to increased mitochondrial outer membrane permeability (MOMP), loss of mitochondrial transmembrane potential and oxidative phosphorylation and release of multiple pro-apoptotic molecules such as second mitochondrial-derived activator of caspases/direct inhibitor of apoptosis binding protein with low pI (Smac/DIABLO), the serine protease HtrA2/Omi and cytochrome c. Smac/DIABLO, as well as HtrA2/Omi, promote apoptosis via interaction with inhibitor of apoptosis proteins (IAPs) [9,10]. IAPs can interact with caspases, inhibit their function and protect cells from apoptosis [11]. Cytochrome c is normally sequestered within the mitochondrial intermembrane space playing an essential role as an electron carrier in the respiratory chain. Once released from mitochondria, cytochrome c associates with apoptotic protease-activating factor 1 (APAF-1), deoxyadenosine triphosphate and procaspase 9, assembling a multiprotein complex called apoptosome. In the apoptosome the initiator procaspase 9 is processed and activated into caspase 9, harboring the proteolytic activity to cleave the effector procaspase 3. Activated caspase 3 subsequently cleaves cellular proteins and promotes the apoptotic machinery (Figure 1) [12].

The permeability of the outer mitochondrial membrane is tightly controlled by members of BCL-2 family proteins via protein-protein interactions. The BCL-2 family of proteins consists of more than 20 different members classified based on the protein structure and key functions into three main subgroups, the anti-apoptotic proteins, the pro-apoptotic multi-domain effector proteins, and pro-apoptotic BCL-2 homology (BH)3 domain-only proteins [13]. Altering the balance between anti-apoptotic and pro-apoptotic BCL-2 proteins can lead to disruption of the apoptotic process and the aberrant survival of cancer cells [14]. The pro-survival BCL-2 proteins comprise B-cell leukemia/lymphoma-2 (BCL-2), myeloid cell leukemia-1 (MCL-1), B-cell lymphoma-extra large (BCL-XL; BCL2L1), B-cell lymphoma-w (BCL-W/BCL2L2), BCL2-like protein 10 (BCL-B/BCL2L10) and BCL-2 related gene A1 (A1/BCL2A1; also known as BFL-1). The pro-survival BCL-2 proteins sequester the pro-apoptotic BCL-2 proteins thereby mediating their anti-apoptotic activity. The anti-apoptotic BCL-2 proteins share a common structure with a transmembrane domain, which is necessary for anchoring to cellular membranes, and four conserved motifs called BH domains (BH1, BH2, BH3, BH4). BH1, BH2, and BH3 domains create a hydrophobic pocket, which is critical for interaction with pro-apoptotic BCL-2 proteins. Interestingly, some proteins of this group have isoforms translated from the same gene that possess pro-apoptotic activity. For example, there are three known isoforms of MCL-1 including full-length MCL-1 (MCL-1L), MCL-1 short (MCL-1S) and MCL-1 extra short (MCL-1ES) generated by alternative splicing of the same gene. In contrast to the anti-apoptotic protein MCL-1L, both MCL-1S and MCL-1ES have different protein structures and display pro-apoptotic activities [15].

Based on their protein structure and key functions, the pro-apoptotic proteins of the BCL-2 family can be divided into following groups: (1) multi-domain effector proteins including BCL-2–associated X protein (BAX), BCL-2 antagonist/killer 1 (BAK1) and BCL-2 homologous antagonist killer (BOK), and (2) BH3 domain-only proteins that comprise BCL-2-interacting mediator of cell death (BIM/BCL2L11), BID, NOXA/PMAIP1, BCL-2 antagonist of cell death (BAD), p53 upregulated modulator of apoptosis (PUMA/BBC3) and harakiri (HRK).

**Figure 2:**
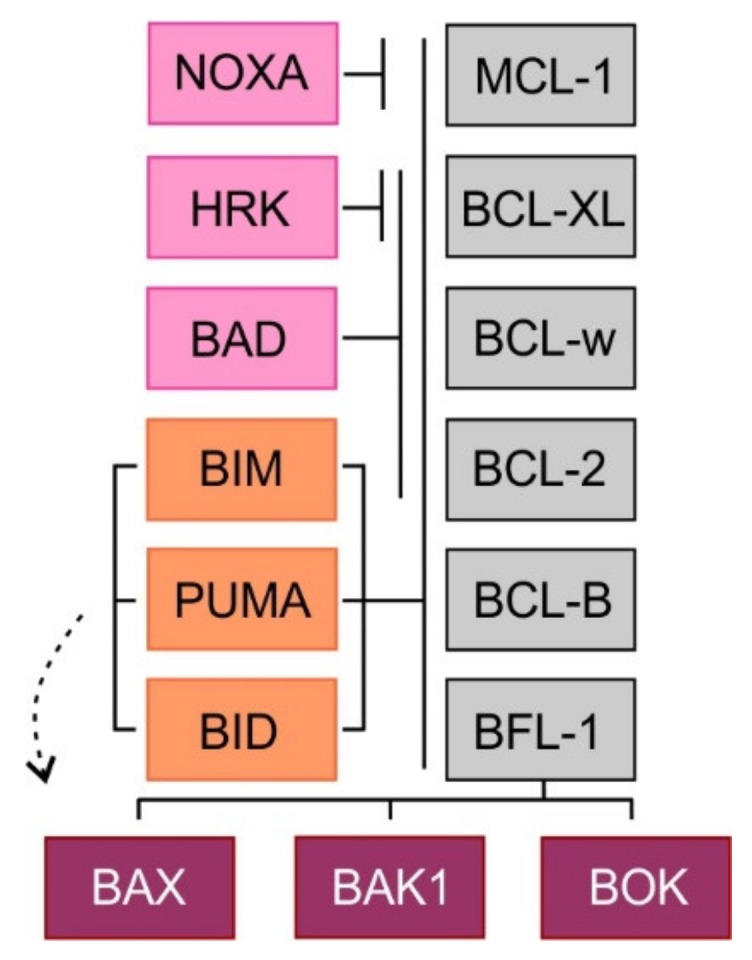
Binding profile of pro-apoptotic BH3 domain-only proteins and multi-domain effector proteins to pro-survival proteins. BH3 domain-only proteins are depicted in orange (direct activators) and pink (sensitizers).

The multi-domain effector proteins BAX, BAK1, and BOK contain a transmembrane domain and three BH3 domains (BH1, BH2, BH3). Activated proteins BAX and BAK form homodimers and heterodimers and create pore-like structures in the outer mitochondrial membrane resulting in its increased permeability for intermembrane space molecules [16]. The precise function of the third member of this family, BOK remains elusive [17]. The so-called BH3 domain-only pro-apoptotic proteins contain only one BH3 domain and some of them also one transmembrane domain. Two models of apoptosis induction have been proposed. In the direct activation model, the BH3 domain-only proteins act either as direct activators of apoptosis (BIM, BID, and perhaps PUMA), possessing the ability to activate the effectors BAX and BAK1, or as sensitizers (NOXA, BAD, HRK). Because the sensitizers cannot directly interact with BAX or BAK1, their pro-apoptotic effect is mediated through binding to anti-apoptotic BCL-2 proteins and subsequent release of BH3 domain-only activators. In the second, indirect model of apoptosis, permanently active forms of BAX and BAK1 are bound to the anti-apoptotic BCL-2 proteins. Increased expression of pro-apoptotic proteins leads to the displacement of these active forms of pro-apoptotic effector proteins, which triggers the mitochondrial apoptosis. Selective binding and different affinities between anti-apoptotic and BH3 domain-only proteins have been shown. While BH3 domain-only activators bind to all anti-apoptotic proteins, BH3 domain-only sensitizers display selective interactions with anti-apoptotic proteins (NOXA-MCL-1, HRK-BCL-XL) caused by differences in the amino acid sequence of the BH3 domain [18] (Figure 2). Importantly, it has been repeatedly demonstrated that cancer cells, whose anti-apoptotic BCL-2 proteins are occupied by BH3 domain-only activators are so-called “primed for death” [18]. Such cells can undergo rapid apoptosis, when exposed to BH3 domain-only sensitizers or BH3 mimetics, a class of anti-tumor molecules that displace BH3 domain-only activators to activate BAX/BAK1 and trigger apoptosis [19].

## 3. B-Cell Non-Hodgkin Lymphomas: Pathogenesis and Classification

**Figure 3:**
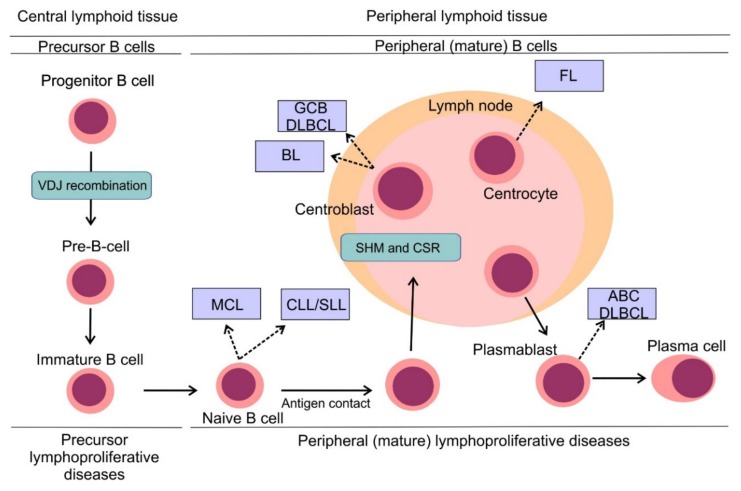
Pathogenesis of B-cell non-Hodgkin lymphomas. Simplified scheme of B cell development showing distinct types of B-NHLs arising from different non-malignant lymphoid counterparts. Reprinted with permission. ^©^ (2020) American Society of Clinical Oncology. All rights reserved. Nogai, H. et al.: J. Clin. Oncol. 29, 2011: 1803–1811 [20].

B-cell non-Hodgkin lymphomas (B-NHLs) represent a heterogeneous group of hematologic malignancies, each arising from different non-malignant lymphoid counterparts (Figure 3) [20]. B cells develop from hematopoietic stem cells in the bone marrow compartment, the primary lymphoid organ and upon their release into peripheral blood, B cells mature in secondary lymphoid tissues including lymph nodes, spleen or tonsils. B cell development comprises different stages, during which lymphocytes undergo critical processes necessary for their proper development, including generation and expression of a functional surface B-cell receptor (BCR). One of the first events to occur is VDJ recombination, the process in which double-strand DNA breaks are randomly introduced by recombinase activating gene *RAG1* and *RAG2* into gene segments encoding variable (V), diversity (D) and joining (J) regions of the BCR with following DNA repair by non-homologous end joining [21]. This process ensures high variability of BCRs on the surface of B-cells capable to face multiple antigens during the immune response [22]. Once the surface BCR is expressed, B cells leave the bone marrow, becoming mature naïve B cells ready to be exposed to various antigens. Another two events modifying the coding sequence of BCR occur in secondary lymphoid tissues: somatic hypermutation (SHM) and class switch recombination (CSR). Both events are mediated by activation-induced cytidine deaminase (AID) [23]. In the case of SHM, AID introduces random mutations into the coding sequence of the variable region of the BCR, which results in a changed affinity for the immunizing antigens. While a randomly increased affinity to antigen would foster the pro-survival signaling from BCR and increase the mitotic activity of the lymphocyte, a decreased affinity would lead to triggering apoptosis and demise of the lymphocyte clone. CSR that enables the switching of the heavy chain class of Ig molecule (e.g., from IgM to IgG) is implemented by DNA recombination. Unfortunately, VDJ recombination, SHM, and CSR are prone to mistakes that can introduce genetic alterations of the developing lymphocytes and contribute to their malignant transformation (Figure 3) [20].

The recent World Health Organization (WHO) classification of lymphoid malignancies identifies approximately fifty mature lymphoproliferative disorders of B-cell origin with distinct clinical, pathological and genetic features [24]. Lymphomas can be divided into aggressive (high-mitotic activity) and indolent (low-mitotic activity) subtypes, which reflects the clinical behavior of these entities. Aggressive lymphomas require immediate treatment, while indolent lymphomas can be subject to watchful waiting in a large proportion of patients. Diffuse large B-cell lymphoma (DLBCL) represents the most common lymphoma subtype and accounts for 30%–40% cases in adults [25]. DLBCL is an aggressive lymphoma subtype requiring treatment upon diagnosis. Two, histologically indistinguishable DLBCL subtypes have been identified by gene expression profiling, each arising from a different cell of origin (COO) [26]. Germinal center B-cell-like (GCB) and activated B-cell-like COO DLBCL subtypes are each driven by distinct oncogenic pathways, display different clinical behavior and have different clinical outcomes, with ABC DLBCL having significantly worse outcome compared to GCB DLBCL [27,28]. Follicular lymphoma (FL) is the second most prevalent subtype of malignant lymphomas and accounts for approximately 20% of all lymphoma cases in adults [25]. It is typically an indolent disease with long term survival. Other frequently diagnosed aggressive B-NHL include mantle cell lymphoma (MCL) and Burkitt lymphoma (BL), while other prevalent indolent lymphomas comprise marginal zone lymphoma (MZL) and small lymphocytic lymphoma (SLL). On a molecular level, SLL refers to the same disease as chronic lymphocytic leukemia (CLL) with specific differences in the clinical picture. CLL is the most common leukemia of the adult in the Western hemisphere but is a rare disease in the Far East. CLL typically presents with hyperlymphocytosis, while the dominant finding in the clinical picture of patients with SLL is lymph node involvement.

## 4. Deregulation of BCL-2 Proteins in B-Cell Non-Hodgkin Lymphomas

Deregulation of mitochondrial apoptosis is a hallmark of lymphomas. Indeed, BCL-2 was originally discovered because of its involvement in translocation *t*(14;18) in follicular lymphoma [29]. Later on, it was discovered that the aberrant expression of BCL-2 protein contributes to the pathogenesis of many types of human malignancies, including leukemias, lymphomas, and cancers. Within B-NHL BCL-2 overexpression commonly arises from genetic abnormalities. Importantly, the frequency of these alterations, as well as the extent of *BCL2* mRNA and protein expressions, are substantially different in distinct lymphoma subtypes (Table 1).

Chromosomal translocation *t*(14;18)(q32;q21), juxtaposing *BCL2* gene (18q) under the control of the immunoglobulin heavy chain gene promoter (14q), leads to constitutive expression of BCL-2, inhibition of apoptosis and extended cell survival [45]. Translocation *t*(14;18)(q32;q21) is a genetic hallmark of follicular lymphoma (FL) and is detected in approximately 90% of all FL cases. The remainder 10% FL cases lack *BCL2* gene translocation and display distinct molecular features with activated B cell-like, NFκB and proliferation expression profiles and frequent lack of BCL-2 protein expression. Interestingly, no differences in overall survival have been shown between translocation-positive and negative FL cases [35]. Genetic alterations (chromosomal translocations, gene amplification, and single nucleotide variants) of *BCL2* genes are frequent abnormalities in DLBCL, however, the frequency of distinct alterations, as well as prevalence of BCL-2 positivity differ between the two major COO subtypes. The translocations *t*(14;18)(q32;q21), detected in more than 30% GCB DLBCL have been associated with high BCL-2 protein expression and poor outcome [33]. Interestingly, this chromosomal aberration is not detected in ABC DLBCL [33,46]. Amplifications of 18q21 locus resulting in BCL-2 overexpression are in contrary significantly more prevalent in ABC DLBCL and are detected in approximately 20% of ABC DLBCL cases [34]. *BCL2* SNVs can be found in approximately 8% of all DLBCL cases and are more frequently detected in the GCB than in the ABC DLBCL subtype [33]. *BCL2* SNVs usually co-occur with *BCL2* translocation, possibly as a consequence of ongoing aberrant somatic hypermutation [47]. SNVs tend to be located in the flexible loop domain of *BCL2* gene, while mutations in BH domains that could impact interaction with BH3 mimetics are rare [47]. Some studies showed that *BCL2* SNVs were associated with shorter progression-free survival while other studies did not [33,47]. In mantle cell lymphoma (MCL), BCL-2 protein is overexpressed in virtually all cases. Similarly to ABC DLBCL, *BCL2* amplification is frequently found in MCL, while the translocations are rare [39]. Another cytogenetic abnormality contributing to high BCL-2 protein expression in MCL is loss of 13q14 locus by deletion [48]. The cluster at 13q14.3 contains genes for two microRNAs, *miR-15a* and *miR-16-1,* both of which negatively regulate *BCL2* at the posttranscriptional level. The loss of this chromosomal region thus results in high BCL-2 expression [49]. Similarly, high BCL-2 protein expression can be documented in virtually all patients with CLL and deletion of 13q14 is common in CLL [43,50,51]. Another mechanism contributing to high BCL-2 expression in CLL is hypomethylation of *BCL2* gene [43]. In contrast to the above mentioned B-NHLs, the level of BCL-2 expression in Burkitt lymphoma is low or undetectable, which has been used as a part of the diagnostic algorithm of this lymphoma subtype [40].

Although the role of other anti-apoptotic proteins in the pathogenesis of B-NHL is less clear, it is probable that various B-NHL subtypes rely on more than one anti-apoptotic protein [52]. The potential role of MCL-1 in lymphoma pathogenesis was demonstrated in transgenic mouse models in which MCL-1 transgenic mice developed B-cell lymphomas at high frequency [53]. Limited data show that MCL-1 protein is highly expressed in aggressive B-NHL, including DLBCL (84% cases), BL (89% cases) and in grade 3 FL (100%). Other B-NHL subtypes (MCL, MZL, SLL) display lower and less frequent MCL-1 positivity [54,55]. Molecular mechanisms leading to aberrant MCL-1 expression have been studied in DLBCL. It has been shown that high MCL-1 expression is more frequent in the ABC subtype. *MCL1* locus (1q21) gain/amplification and constitutive activation of the STAT3 pathway were identified as key drivers of aberrant MCL-1 expression in this lymphoma subtype [56].

Although BCL-XL appears to be frequently expressed, its overexpression responsible for resistance to specific BCL-2 inhibitors has been observed in response to pro-survival signaling from the lymph node microenvironment rather than genetic alterations. Moreover, BCL-XL is critical for the proper development of platelets, and targeted inhibition of BCL-XL has been associated with thrombocytopenia (see Section 5.). Another anti-apoptotic protein, BCL-W has been shown to be overexpressed in the majority of aggressive and indolent lymphoma, including DLBCL (equally in both COO subtypes), BL, MCL, FL, and MZL, making it a potential therapeutic target in B-NHL [52].

Although overexpression of pro-survival BCL-2 proteins as a consequence of genetic alterations is a frequent event in B-NHL, the delicate balance between anti-apoptotic and pro-apoptotic BCL-2 proteins might be disrupted by lack of pro-apoptotic BCL-2 proteins, leading to apoptosis evasion and survival advantage. The key role of pro-apoptotic protein BIM in the pathogenesis of MCL has been proposed in a transgenic mouse model, when cyclin D1-transgenic mice harboring BIM-deficient B cells developed lymphomas with histopathologic and molecular features of human MCL [57]. Although approximately one-third of currently available MCL cell lines harbor mono- or biallelic *BIM/BCL2L11* deletion, we have shown that this alteration is rarely found in patients with newly diagnosed MCL [58]. Even though a complete lack of BIM protein expression as a result of gene deletion is probably rare, the level of BIM protein expression was shown to negatively correlate with prognosis in MCL [59].

Besides frequent alterations of genes encoding BCL-2 proteins, lymphomas may evade apoptosis through activated signaling pathways, resulting in transcriptional deregulation of BCL-2 proteins. Deregulation of the phosphate and tensin homolog (PTEN)-phosphatidylinositol 3-kinase (PI3K) pathway plays an important role in the pathogenesis of GCB DLBCL. Loss of *PTEN*, either by gene deletion or amplification of miR-17-92, which suppresses *PTEN*, can be found in approximately 55% GCB DLBCL [60]. Loss of *PTEN* leads to activation of PI3K-AKT pathway and upregulation of downstream targets, including e.g., transcription factor MYC, thus promoting growth, survival, and proliferation [60]. It has been shown that Akt-mediated phosphorylation inhibits the pro-apoptotic protein BAD. Akt phosphorylates BAD at serine 112 resulting in its dissociation from BCL-2 to form a complex with the 14-3-3 adaptor protein, which is associated with cell survival [61]. On the contrary, constitutive activation of NFκB pathway is a hallmark of ABC DLBCL and several studies have shown the dependence of ABC DLBCL on NFκB signaling [62,63]. NFκB is a family of related transcription factors that are normally kept inactive in the cytoplasm by interaction with specific inhibitors. Stimulation through various receptors, including the surface B-cell receptor, results in the release of NFκB factors, their activation, translocation to the nucleus and transcription of target genes. Three upstream molecules CARD11, BCL-10/BCL10 and MALT-1, forming a CBM complex, are necessary to convert signals from the BCR into the cell to activate the so-called canonical NFκB pathway [63]. Missense mutations of *CARD11*, leading to strong activation of NFκB signaling, are frequent events in both GCB and ABC DLBCL [33,64]. Another pathogenic mutation identified a few years later was a gain of function mutation of *MYD88*, an adaptor protein that mediates toll and interleukin (IL)-1 receptor signaling and activates NFκB pathway in distinct lymphoma subtypes. *MYD88* (*L265P*) can only be detected in ABC DLBCL and in a subset of MZL called mucosa-associated lymphoid tissue (MALT) lymphoma [65]. Other well-defined molecular events deregulating the CBM complex have been reported in MALT lymphomas. Chromosomal translocations t(14;18)(q32;q21) and t(1;14)(p22;q32), bringing *MALT1* and *BCL10* under the control of the immunoglobulin heavy chain promoter have been associated with this lymphoma subtype. Both *MALT1* and *BCL10* translocations can make CBM complex formation independent of upstream signaling, resulting in constitutive NFκB pathway activation [66,67]. The third translocation involved in aberrant NFκB signaling in this lymphoma subtype is the t(11;18)(q21;q21), resulting in the expression of a chimeric IAP2-MALT-1 protein. IAP2-MALT-1 can interact and deregulate components of a multiprotein IκB-kinase (IKK) complex, resulting in NFκB pathway activation [68]. Known NFκB targets that promote cell survival via inhibition of apoptosis include anti-apoptotic BCL-2 proteins BCL-XL and A1, caspase inhibitors from the IAP family and Cellular FLICE (FADD-like IL-1β-converting enzyme)-inhibitory protein (c-FLIP) [69].

Another important mechanism of how lymphomas evade apoptosis is the deregulation of the DNA damage pathway. *TP53* alterations (deletions, mutations) are recurrently found in aggressive lymphomas but are rather rare in indolent lymphomas [70]. Prognostically, *TP53* aberrations have repeatedly correlated with adverse outcome and chemoresistance [71,72,73]. In indolent lymphomas, *TP53* aberrations have been associated with the transformation to aggressive lymphomas [74,75]. P53 directly transactivates pro-apoptotic BH3-only proteins PUMA and NOXA [76,77]. As a consequence of structural or functional p53 inactivation, the critical DNA damage response is disrupted, which increases genomic instability and facilitates the survival of lymphoma cells in response to genotoxic cytostatics.

## 5. Therapeutic Inhibition of Anti-Apoptotic BCL-2 Proteins

Given the fact that anti-apoptotic BCL-2 proteins play a crucial role in lymphoma pathogenesis, disease progression and drug resistance, the efforts to target them therapeutically have been underway for several decades. Moreover, therapeutic targeting of BCL-2 proteins is upstream and, therefore, independent of *TP53*, the key tumor suppressor gene frequently mutated in distinct lymphoid malignancies, overcoming the negative prognostic impact of this genetic alteration. Due to its indisputable role in the pathogenesis of B-NHL, BCL-2 protein has been in the spotlight of extensive efforts to develop targeted therapies. Several approaches have been explored to target this protein, including antisense oligonucleotides inhibiting expression of BCL-2 protein, peptide inhibitors or small molecule inhibitors.

Oblimersen sodium (G3139, Genansense, Genta Inc, Berkeley Heights, NJ, USA) was the first BCL-2 inhibitor tested in clinical practice. Oblimersen sodium is a phosphorothioated 18 basis DNA molecule, which was designed to complementary bind to the first six codons of *BCL2* mRNA leading to *BCL2* mRNA degradation and decrease in BCL-2 protein expression [78]. Despite evidence of efficacy in early clinical trials in hematological malignancies including CLL, the phase III trial combining oblimersen sodium with (immuno-) chemotherapy in patients with relapsed or refractory CLL failed to show clear clinical benefit and oblimersen sodium has never been approved by the US Food and Drug Administration (FDA) [79].

Another attempt to directly target anti-apoptotic BCL-2 proteins was made with small molecule inhibitor, obatoclax (GX-15-070). Obatoclax binds to all key anti-apoptotic proteins, BCL-2, BCL-XL and MCL-1 resulting in the displacement of pro-apoptotic proteins BAX, BAK or BIM and apoptosis induction [80]. Other mechanisms of action of obatoclax have been described including S/G2 cell cycle arrest resulting in growth inhibition. These observations indicate that besides anti-apoptotic BCL-2 proteins obatoclax has other targets [81]. Obatoclax has been tested in several phase 1-2 clinical trials in various types of B-NHL and CLL. These trials showed modest clinical activity of obatoclax as a single agent and did not confirm the synergistic effect of obatoclax with various anti-lymphoma agents suggested in preclinical studies. Moreover, neurological toxicity has been observed in all these trials [82,83,84,85]. Both, the modest clinical activity as well as the toxicity profile limited development of obatoclax as a therapeutic agent in B/NHL and CLL.

A breakthrough in the development of BCL-2 inhibitors was achieved with ABT-737, a small molecule inhibitor of BCL-2, BCL-XL, and BCL-W. ABT-737 displays a much higher binding affinity to anti-apoptotic proteins than obatoclax, with subsequent release of proapoptotic proteins and BAX/BAK1-dependent apoptosis induction [86]. ABT-263 (Navitoclax, Abbott Laboratories, North Chicago, IL, USA) is a chemically closely related agent with a similar binding affinity to BCL-2 proteins, which is orally available [87]. The promising preclinical activity led to the clinical testing of Navitoclax in various B-NHL and CLL. Phase 1 study of navitoclax in patients with relapsed or refractory lymphoid malignancies demonstrated clinical activity across all histological subtypes with the best activity seen in CLL [88]. A follow-up phase 1 dose-escalation study of single-agent navitoclax in patients with relapsed or refractory CLL confirmed the efficacy of navitoclax with durable responses and median progression-free survival of 25 months in heavily pretreated CLL patients [89]. The dose-limiting toxicity of navitoclax observed in these trials was thrombocytopenia. Rapid reduction in platelet count (grade 3–5) was observed in 28% of patients and prevented the exploration of higher doses of navitoclax [89]. Thrombocytopenia was caused by inhibition of BCL-XL in platelets, that was shown to be dependent on BCL-XL for survival [90,91]. Further phase 2 trial of navitoclax in combination with rituximab vs rituximab alone in patients with previously untreated CLL demonstrated improved objective response rate (ORR) of rituximab plus navitoclax vs rituximab alone (50% vs 35%, respectively), with a further increase of ORR (70%) when navitoclax with rituximab was administered until progression [92]. It has been shown that CLL patients with del(17p) or high levels of BCL-2 had significantly better clinical responses when treated with navitoclax [92]. This observation together with the navitoclax-induced thrombocytopenia caused by BCL-XL inhibition led to efforts to develop a BCL-2 specific inhibitor.

**Figure 4:**
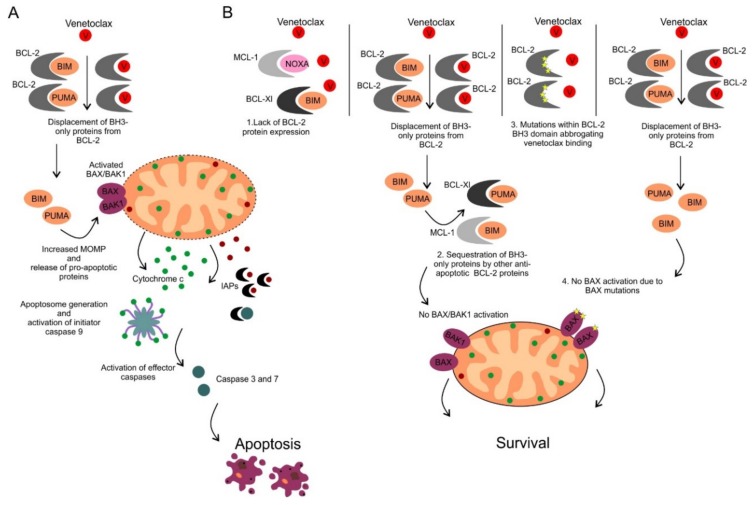
(**A**) Mechanism of action of venetoclax. Venetoclax binds to the BH3 domain of BCL-2 protein with subsequent release of proapoptotic proteins, activation of BAX/BAK1 and apoptosis induction. (**B**) Mechanisms of resistance to venetoclax. Four different mechanisms of inherited or acquired resistance to venetoclax include: 1. Lack of BCL-2 protein expression, 2. Overexpression of other anti-apoptotic BCL-2 proteins that sequester pro-apoptotic BH3-only proteins displaced from BCL-2 protein following its pharmacological inhibition by venetoclax, 3. Acquired mutations within the BH3 domain of BCL-2 abrogating venetoclax binding, 4. Acquired mutations of *BAX* preventing BAX activation and apoptosis induction.

Venetoclax (ABT-199/GDC-0199) was the first highly selective orally available BCL-2 inhibitor developed by the re-engineering of navitoclax [93]. Similarly to navitoclax, venetoclax displays high affinity for BCL-2 (K_i_ < 0.010 nM), however its binding affinity to other anti-apoptotic proteins including BCL-XL (K_i_ = 48 nM), MCL-1 (K_i_ > 444 nM) and BCL-W (K_i_ = 245 nM) is much weaker [93]. The mode of action of venetoclax is shown in Figure 4A. Preclinical in vitro and in vivo studies in various human hematological cell lines and xenograft models demonstrated significant anti-tumor efficacy which led to early clinical testing [93].

## 6. Venetoclax in Chronic Lymphocytic Leukemia/Small Lymphocytic Lymphoma

The first-in-human study of venetoclax was conducted in patients with relapsed or refractory CLL. A single dose of venetoclax (200 mg or 100 mg) in two out of three patients in the first cohort resulted in prompt regression of palpable lymphadenopathy and more than 95% reduction in peripheral lymphocyte count. All three patients in the first cohort developed laboratory signs of tumor lysis syndrome (TLS) within 24 h after a single dose of venetoclax indicating prompt anti-leukemic effect [93]. This multicenter phase 1 clinical trial finally enrolled 116 patients with relapsed or refractory CLL, 56 patients in the dose-escalation cohort and an additional 60 patients in the expansion cohort. In the dose-escalation cohort, clinical TLS occurred in 3 out of 56 patients with one death and one acute renal failure requiring hemodialysis. The dosing schedule was further adjusted to the final weekly stepwise ramp-up in doses (20 mg, 50 mg, 100 mg, 200 mg, 400 mg daily), with the initial dose of 20 mg daily to the maximum dose of 400 mg daily, in the expansion cohort. No clinical TLS was observed in the expansion cohort. Other toxicities included mild diarrhea (in 52% of the patients), upper respiratory tract infection (in 48%), nausea (in 47%), and grade 3 or 4 neutropenia (in 41%). Pooled data from both cohorts showed ORR of 79% with complete response (CR) in 20% of patients. Importantly, similar results were achieved in patients with 17p deletion and IGHV unmutated status, the known adverse prognostic factors in CLL [94]. Similar results were confirmed in phase 2 single-arm trial of venetoclax monotherapy in patients with relapsed refractory CLL with 17p deletion. Venetoclax was administered once daily with the same weekly dose ramp-up schedule and given until disease progression. The primary endpoint of the study, ORR was achieved in 79.4% patients, which led to the accelerated approval of venetoclax for CLL patients who relapsed or are refractory to at least one prior line of therapy by the FDA in April 2016 and by the European Medicines Agency (EMA) in December 2016 [95].

A further step was to evaluate the safety and efficacy of venetoclax in combination with anti-CD20 antibodies Rituximab (R) and later, Obinutuzumab (GA101; G). Phase 1b dose-escalation study of single-agent venetoclax in combination with Rituximab in patients with relapsed or refractory CLL or SLL showed acceptable safety and high efficacy, with ORR of 86% and CR or CR with incomplete count recovery (CRi) rate of 51% [96]. Subsequently, a phase 3 trial of venetoclax (up to two years) in combination with Rituximab (for the first 6 months) compared to bendamustine plus rituximab for 6 months demonstrated ORR and CR rate of 93.3% and 26.8% vs 67.7% and 8.2%, respectively. Importantly, R-Venetoclax induced minimal residual disease (MRD) negativity in 62.4% of patients compared to 13.3% in the R-Bendamustine arm. The primary endpoint of this trial was met as significantly higher PFS in R-Venetoclax compared to R-Bendamustine (2-year investigator-assessed PFS rate 84.9% vs 36.3%, respectively) was demonstrated [97]. The results of this trial led to the FDA and EMA approval of Venetoclax in relapsed or refractory CLL in June 2018 and September 2018, respectively. Subsequently, phase 1b trial of Venetoclax in combination with G in patients with relapsed or refractory or previously untreated CLL patients demonstrated an acceptable safety profile. The most common grade 3–4 adverse event was neutropenia which occurred in 58% and 53% of relapsed refractory and previously untreated patients, respectively. Rates of grade 3–4 infections were 29% and 13% in relapsed refractory and previously untreated patients, respectively. No fatal infections occurred in previously untreated CLL patients. High overall best response rates and CR rates were observed in relapsed refractory (95% and 37%) and previously untreated (100% and 78%) CLL patients [98]. Based on this experience a phase 3 trial of venetoclax in combination with Obinutuzumab (GA101; G) vs chlorambucil with G in previously untreated CLL patients with coexisting conditions (Cumulative Illness Rating Scale greater than 6 or creatinine clearance lower than 70 mL per minute) was conducted. In this trial, both venetoclax and chlorambucil were given for a fixed duration of 12 cycles. Obinutuzumab was administered for 6 cycles in both arms. In this trial significantly higher PFS rate was observed in the G-Venetoclax compared to G-Chlorambucil (2-year PFS 88.2% vs 64.1%, respectively). The benefit of G-Venetoclax over G-Chlorambucil was observed across all prognostic subgroups, including patients with *TP53* alteration or unmutated IGHV status. No new safety signals or higher incidences of known toxic effects were observed. The adverse events in both treatment arms were similar in severity, and significant differences were detected only in the incidence of metabolic disorders and gastrointestinal disorders. The number of fatal adverse events was higher in the G-venetoclax arm than in the G-chlorambucil arm 7.5% vs. 3.7% [99]. Based on this trial FDA granted approval of venetoclax for patients with CLL or SLL in May 2019.

## 7. Venetoclax in B-Cell Non-Hodgkin Lymphomas

Venetoclax monotherapy was also tested in patients with various types of relapsed or refractory B-NHL. A total of 106 patients with relapsed or refractory B-NHL (mostly DLBCL, FL, and MCL) were enrolled in a phase 1 trial. Venetoclax was well tolerated, the most common adverse events were gastrointestinal toxicity and neutropenia. In contrast to previous experience in CLL, clinical TLS was not observed in the B-NHL population. The activity of venetoclax varied among B-NHL subtypes, with the highest efficacy observed in MCL (ORR 75%; CR, 21%). Even though BCL-2 is overexpressed in all FL cases, the ORR and CR rates in this lymphoma subtype were only 38% and 14%, respectively. In DLBCL the ORR and CR rates were also disappointing and reached only 18% and 12%, respectively [38]. Given the modest activity of single-agent venetoclax in B-NHLs, venetoclax has been studied as part of combinatorial regimens, usually with well-established immunochemotherapy such as R-CHOP (Rituximab, Cyclophosphamide, Doxorubicin, Vincristine, and Prednisone), dose-adjusted (DA)-EPOCH-R (etoposide, prednisone, vincristine, cyclophosphamide, doxorubicin, and rituximab) or R-Bendamustine. For example, the phase 1b/2 Cavalli trial of venetoclax in combination with R or G plus CHOP was conducted in patients with B-NHL (most patients had previously untreated DLBCL or FL). The phase 1b part of the trial established the phase 2 dose for venetoclax with R-CHOP (21-day cycle) at 800 mg days 4 to 10 of cycle 1 and days 1 to 10 of cycles 2 to 8. The main safety signal in this trial was the higher rate of neutropenia, febrile neutropenia and thrombocytopenia as compared to historical data with R-CHOP alone [100]. The phase 2 part of the trial (venetoclax plus R-CHOP) was conducted only in newly diagnosed DLBCL patients and the results were compared to matched historical controls from the GOYA phase 3 trial (R-CHOP treated patients) [27]. A higher rate of toxicity was observed for the venetoclax plus R-CHOP arm, especially driven by the higher rate of hematological toxicity and infections. Although the end of treatment CR rates (the primary endpoint) did not differ significantly between these two cohorts, venetoclax plus R-CHOP appeared to improve CR rates in BCL-2 positive disease and especially in double-hit lymphomas [101]. Currently, a phase 2/3 trial of venetoclax plus R-CHOP or DA-EPOCH-R vs R-CHOP or DA-EPOCH-R alone in patients with DLBCL or high-grade B-NHL with translocations of *BCL2* and *MYC* genes (double-hit lymphomas) or dual expression of BCL-2 and MYC proteins (double expressing lymphoma) is ongoing (NCT03984448). The efficacy and safety of venetoclax in combination with R or with R-Bendamustine, as well as R-Bendamustine was evaluated in the phase 2 trial in relapsed or refractory FL (NCT02187861). Similar results in terms of efficacy were observed in venetoclax plus R-Bendamustine (68% ORR with 50% CR) compared to R-Bendamustine (64% ORR with 41% CR). The ORR and CR rates were only 33% and 14% in the chemotherapy-free arm, although the majority of the patients in this arm were refractory to the last treatment [102]. In previously untreated FL, combinations of venetoclax plus G (NCT02877550) or venetoclax plus G-bendamustine (NCT03113422) are currently being tested. The combination of venetoclax plus the Bruton’s tyrosine kinase BTK inhibitor ibrutinib (given until progression) was evaluated in a pivotal single-arm phase 2 study in patients with relapsed or refractory MCL (23 out of 24 patients) or previously untreated MCL with contraindication for cytotoxic chemotherapy (1 out of 24 patients). The CR rate at week 16 as assessed by CT (primary endpoint) was 42%, which was higher compared to historical control of 9% at this time point with ibrutinib monotherapy. PET-based CR rate and ORR at week 16 was 62% and 71%, respectively. Moreover, 78% of the patients had an ongoing response at 15 months [103]. Currently, a phase 3 trial of ibrutinib plus venetoclax vs ibrutinib plus placebo in patients with relapsed or refractory MCL is ongoing (NCT03112174, SYMPATICO trial). In the experimental extension of the SYMPATICO study newly diagnosed elderly patients or younger patients with *TP53* mutation are being enrolled for frontline therapy with the combination of ibrutinib and venetoclax (this is not placebo-controlled arm). Other two-drug combinations with venetoclax for newly diagnosed MCL patients include immunomodulatory agent lenalidomide (NCT03523975) or bendamustine (NCT03872180), in each case with anti-CD20 antibody (rituximab or obinutuzumab, respectively). Promising results were shown in a recent phase 2 study of single-agent venetoclax (given for maximun of two years) in patients with previously treated Waldenstrom macroglobulinemia (a rare B-cell lymphoproliferative disorder characterized by secretion of IgM molecules). ORR was 87% and 2-year PFS reached 76%. These encouraging results were achieved even though the enrolled patients were heavily pretreated (up to 10 previous lines of therapy), including 52% of patients, who were previously treated with ibrutinib [104].

## 8. Selective Inhibitors of MCL-1 and BCL-XL Inhibitors in Chronic Lymphocytic Leukemia and B-Cell Non-Hodgkin Lymphomas

Apart from BCL-2 inhibitors, therapeutic targeting of MCL-1 has recently become a promising treatment strategy in B-NHL. Given the indisputable role of MCL-1 in pathogenesis of B-NHL (discussed above) and also in resistance of B-NHL to anti-lymphoma agents including venetoclax (see below), MCL-1 represents an attractive therapeutic target. Targeting MCL-1 directly with selective inhibitors, or indirectly with agents that cause downregulation of MCL-1 as part of their mechanism of action, proved to be efficient in numerous preclinical models of B-NHL, including DLBCL, MCL, BL or CLL [105,106,107,108,109]. Interestingly, we and others have shown that combined inhibition of MCL-1 and BCL-2 is highly effective in preclinical models of B-NHL [58,105,106,107]. Selective MCL-1 inhibitors have also entered early clinical testing in various hematologic malignancies. MCL-1 inhibitors currently tested in phase 1 trials (alone or in combination with venetoclax) in relapsed or refractory B-NHL include MIK665 (also known as S64315, NCT02992483), AMG176 (NCT03797261, currently suspended to evaluate safety), AMG397 (NCT03465540) and AZD5991 (NCT03218683).

Although BCL-XL represents a well-validated therapeutic target, the clinical use of BCL-XL inhibitors (such as ABT263) was limited due to thrombocytopenia caused by inhibition of BCL-XL in platelets (see above). Interestingly, Khan et al. used PROTAC (proteolysis-targeting chimera) technology to avoid BCL-XL inhibition in platelets. By converting ABT263 into DT2216, Khan et al. developed a BCL-XL PROTAC, that targets BCL-XL to the Von Hippel Lindau (VHL) E3 ligase for degradation by the proteasome. Because VHL expression is low in platelets, DT2216 was less toxic to platelets. DTT2216 showed in vitro and in vivo efficacy in various preclinical models of hematological malignancies [110].

## 9. Determinants of Sensitivity and Resistance to BCL-2 Inhibition by Venetoclax

Because BCL-2 inhibitor venetoclax is the only agent that has already been approved for the treatment of patients with hematologic malignancies, we will focus only on mechanisms of sensitivity/resistance to BCL-2 inhibition by venetoclax.

Since the discovery of venetoclax (originally ABT-199) in 2003, it has been proposed that BCL-2 protein expression is a strong determinant of sensitivity to venetoclax (Figure 4B). Although the anti-tumor activity of venetoclax was demonstrated across a panel of B-NHL cell lines, cell lines with high BCL-2 protein expression were significantly more sensitive to venetoclax compared to those with low BCL-2 protein expression [93]. These findings were confirmed later by our group and others in preclinical in vitro and in vivo models of lymphomas [105,111]. Interestingly, similar observations have been shown in the clinical grounds. In a phase 1b/2 trial of venetoclax combined with Rituximab or Obinutuzumab plus CHOP in previously untreated DLBCL, venetoclax combined with R-CHOP improved efficacy in BCL-2 positive DLBCL (including patients with double-expressor and double-hit DLBCL) compared to matched historical controls [100]. Results from a randomized phase II/III trial of venetoclax plus immunochemotherapy (R-CHOP or DA-EPOCH-R) vs immunochemotherapy alone in patients newly diagnosed DLBCL/high-grade B-NHL with translocation of *BCL*2 and *MYC* or dual expression of BCL-2 and MYC proteins are eagerly awaited.

Although important, BCL-2 protein expression is not the only factor determining sensitivity/resistance to venetoclax. Even though follicular lymphoma is a lymphoma subtype with high BCL-2 expression in virtually all cases, anti-lymphoma efficacy of single-agent venetoclax this lymphoma subtype was rather disappointing (see above). It has been shown that it is not solely the expression of BCL-2, but predominantly occupation of BCL-2 by pro-apoptotic proteins that determine sensitivity to venetoclax (Figure 4B). For instance, BCL-2 in CLL and MCL cells is occupied by the pro-apoptotic activator BIM that can be immediately released upon venetoclax exposure and trigger cell death [112]. We and others have shown that cells harboring BCL-2 proteins occupied by BH3 domain-only activators, such as BIM, undergo rapid apoptosis when exposed to BH3 mimetics, and are so-called primed for death [18,105]. The occupational status of BCL-2 can be nowadays tested in cell lines e.g., by a method called BH3 profiling [18]. Interestingly, preclinical data on DLBCL cell lines suggest that dependency on anti-apoptotic proteins, assessed by BH3 profiling, is altered following exposure to CHOP or CHOP compounds underlining the potential of BH3 profiling in predicting therapy outcomes [113]. However, the applicability of this method in the clinical practice, especially in lymphomas with no bone marrow/peripheral blood involvement, remains questionable [18].

Once pro-apoptotic proteins are released from BCL-2 protein targeted by venetoclax, these proteins can promote apoptotic cell death such as in the scenario described above or they can be sequestered by other anti-apoptotic proteins that are not targeted by venetoclax. These proteins serve as a buffer for released pro-apoptotic proteins and might confer resistance to venetoclax (Figure 4B). Several studies have shown that high BCL-2/MCL-1 or BCL-2/BCL-XL ratios are associated with sensitivity to BH3 mimetics in various types of lymphoma [114,115]. It has been shown that DLBCL cell lines harboring amplification of *BCL-*2 and *NOXA/PMAIP1* genes are more sensitive to venetoclax-induced apoptosis. NOXA is a BH3-only protein that can bind and trigger proteasome-mediated MCL-1 degradation, possibly explaining increased sensitivity of cell lines harboring *NOXA/PMAIP1* gene amplifications to venetoclax [116]. Genetic alteration of *NOXA/PMAIP1* is a rare event in DLBCL and cannot be used as a useful biomarker to predict sensitivity to venetoclax. However, pharmacologic induction of NOXA, e.g., by proteasome inhibitors bortezomib or carfilzomib, or 5-azacytidine, might sensitize lymphoma cells to venetoclax-induced apoptosis [116,117].

Lymphoma cell-extrinsic factors can also contribute to acquired resistance to venetoclax. It has been repeatedly demonstrated that CLL or MCL cells cocultured on CD40L-expressing fibroblast feeder cells develop resistance to venetoclax mediated by marked upregulation of BCL-XL [118]. Lymphoma cells often proliferate in hostile niches characterized by hypoxia, acidosis, and lack of nutrients. Changes in cell energy metabolic pathways have been recently reported to contribute to acquired venetoclax resistance [119]. The report may suggest a more complex pattern of adaptation changes as a result of the selective pressure of this mitochondria-targeting agent in addition to the deregulation of BCL-2 family members.

Study of clonal evolution associated with acquired resistance to venetoclax in patients, who experience lymphoma relapse after temporary remission, can largely contribute to our understanding of the mode of action of venetoclax, identify relevant predictive factors and prioritize candidate molecules for more effective drug combinations. Acquired mutations of BCL-2 or *BAX* has been reported to confer venetoclax resistance in vitro and in vivo (Figure 4B). Continuous exposure of lymphoma cells to venetoclax resulted in the selection of clones with missense mutations of *BCL2* BH3 domain thereby abrogating venetoclax binding and conferring drug-resistant phenotype [120]. Other studies reported acquired mutations in the transmembrane domain of the pro-apoptotic *BAX*, which disrupted BAX anchoring to mitochondria and blocked venetoclax–induced apoptosis [120]. Similar findings were confirmed in the clinical setting in CLL patients who became refractory to continuous venetoclax treatment. Mutations in *BCL2* were found to be a frequent event in CLL patients becoming refractory to continuous venetoclax treatment. The identified *BCL*2 mutations (eg., G101V) interfered with venetoclax binding to BCL-2 [121,122,123].

## 10. Conclusions

Deregulation of mitochondrial apoptosis plays a crucial role in pathogenesis, disease progression and drug resistance of B-NHL. Therapeutic targeting of anti-apoptotic BCL-2 proteins is a promising treatment strategy for distinct B-NHL subtypes. Venetoclax, the highly specific small-molecule inhibitor of anti-apoptotic BCL-2 protein, has been approved for the treatment of CLL and is in advanced clinical testing in other B-NHL subtypes. In contrast to CLL, where venetoclax demonstrated outstanding activity, the efficacy of venetoclax in other B-NHL subtypes is heterogeneous and requires further clinical testing and development of new biomarkers predicting sensitivity to this agent.

## Figures and Tables

**Table 1:** Mechanisms of B-cell leukemia/lymphoma-2 (BCL-2) overexpression in B-cell non-Hodgkin lymphomas (B-NHL).

B-NHL Subtype	BCL-2 Positivity *	Mechanism of BCL-2 Overexpression
DLBCL	49%–67% [30,31,32]	*BCL2* translocation in GCB DLBCL (30% of cases) [33] *BCL2* amplification in ABC DLBCL (20% of cases) [34]
FL	> 90% [35,36,37]	*BCL2* translocation (90% of cases) [35,36]
MCL	BCL-2 positive [38]	13q14.3 loss (55% of cases) [39] 18q21 gains (20% of cases) [37,39]
BL	BCL-2 negative, weak expression in up to 20% [40]	NA
CLL/SLL	BCL-2 positive (high expression in majority of the cases) [41]	13q14.3 loss (68% cases) [42] *BCL2* gene hypomethylation [43] *BCL2* translocation (rare, < 5%) [41]
MZL	>80% [44]	unknown

* Different methodologies and different cutoff values defining BCL-2 positivity were used in the following studies.
